# Gaming Disorder Symptom Questionnaire: The Development and Validation of a Screening Tool for ICD-11 Gaming Disorder in Adolescents

**DOI:** 10.3389/fpsyt.2022.848157

**Published:** 2022-03-24

**Authors:** Lina Zhang, Tao Luo, Wei Hao, Yuanyuan Cao, Ming Yuan, Yanhui Liao

**Affiliations:** ^1^National Clinical Research Center for Mental Disorders, Department of Psychiatry, The Second Xiangya Hospital of Central South University, Changsha, China; ^2^Department of Psychology, The First Affiliated Hospital of Nanchang University, Nanchang, China; ^3^Department of Teaching and Research, No. 41 Middle School Urumqi, Urumqi, China; ^4^Department of Psychiatry, Sir Run Run Shaw Hospital, School of Medicine Zhejiang University, Hangzhou, China

**Keywords:** gaming disorder, the Gaming Disorder Symptom Questionnaire-21, scale development, validation, ICD-11

## Abstract

**Background:**

Gaming disorder (GD) has been recognized as an official diagnostic entity in the latest revision of the International Classification of Diseases (ICD-11). However, the majority of previous studies used different instruments, which are not fully consistent with the concept of GD in ICD-11. The development of a screening assessment instrument based on ICD-11 for this new disease entity is very urgent and important.

**Methods:**

The ICD-11 Gaming Disorder Symptom Questionnaire (GDSQ), based on the ICD-11 diagnostic guidelines for GD, was developed by a team of GD experts. A total of 7,790 adolescents were included in this study. Criterion validity was assessed by GDSQ, Video Gaming Dependency Scale (VGDS), weekly game playing time, weekly game video viewing time, and monthly money spent on games. Item structure was measured by factorial analysis. Discrimination between GD and non-GD was examined based on the receiver characteristic curve (ROC).

**Results:**

The GDSQ was very well described by three symptoms of GD (i.e., impaired control, increasing priority to gaming, and continued use despite the occurrence of negative consequences). The internal consistency was excellent (Cronbach's α = 0.964) with good criterion validity and good discriminatory power. The optimal cutoff point for determining the profile of gamers was found to be ≥62 points. The GDSQ revealed that the prevalence of GD was 2.27% in this adolescent sample.

**Conclusion:**

The ICD-11–based GDSQ is a successfully validated measurement scale for GD among adolescents. This study provides a new tool (GDSQ) for us to effectively identify individuals with risk of GD in medical and non-medical settings.

## Introduction

Gaming disorder (GD) has become a significant public health concern. According to market analysis, there were 2.8 billion online game players worldwide and the global gaming market generated $175.8 billion in revenue in 2021 (1). There were 509 million online game players in China until June 2021 (2). With the increasing population of game users, the psychological and physical harm due to excessive gaming behaviors has caused concerns in psychiatry, public health, education, and administration (3, 4). Until now, the mainstream view considers excessive and uncontrollable gaming behavior as an addictive disorder (namely, GD). Adolescents are particularly vulnerable to GD (5, 6) and often experience a series of negative consequences, including low self-esteem, intense negative mood states (e.g., sadness, irritability, and boredom) (7), relationship conflicts, and problems at work or school (8–11).

Plenty of studies have been conducted to assess the prevalence of GD and gaming related problems. However, the lack of a unified instrument among these studies resulted in widespread inconsistency in the estimation of the prevalence rates. For example, studies revealed the prevalence of GD ranged from 3.5 to 17% in China (12) and from 0.3 to 4.9% in the United States (13). Therefore, it is indispensable to establish a set of more effective diagnostic criteria and screening tools for GD.

In 2013, the Diagnostic and Statistical Manual of Mental Disorders (DSM-5) included Internet Gaming Disorder (IGD) in the “Conditions for Further Study” section and proposed a set of tentative diagnostic criteria for IGD (14): indicated by five or more of the nine items (preoccupation, withdrawal, tolerance, unsuccessful attempts to control, loss of life interests, continuation despite problems, deception, escape, and jeopardizing important life aspects) for 12 months (15). It encouraged researchers to utilize the same standard to recognize people with IGD and develop screening instruments.

Nonetheless, the proposed diagnostic criteria for IGD in DSM-5 have some limitations. First, it is questionable whether certain criteria (e.g., preoccupation, tolerance, withdrawal response, and deception) have sufficient sensitivity and specificity or not (16–20). Second, all diagnostic criteria for IGD are equally weighted when counting how many of them are met, but this approach is flawed because it fails to distinguish well between core and non-core symptoms (21). Besides, the consensus about the diagnostic cutoff value for IGD (five of nine items) seems not established. By diagnosing IGD with the Nine-Item Internet Gaming Disorder Scale-Short Form (IGDS9-SF) test, the cutoff point score of 36 in Pontes' study is higher than the optimal cutoff point of 32 in Qin's study (with a sample of 3,742 from Chinese universities and 131 from Chinese clinical settings) (22, 23).

The 11th revision of the International Classification of Diseases (ICD-11) included GD as a disease entity in 2019. The WHO Working Group has proposed the diagnostic guidelines for GD (24, 25), which define the core symptoms of GD as (1) impaired control over gaming behavior (e.g., onset, frequency, intensity, duration, termination, and context); (2) increasing priority to gaming over other life interests and daily activities; and (3) continuation or escalation of gaming activities despite the occurrence of negative consequences. Because of the short history of ICD-11, until now, few research studies have been conducted based on these criteria. Some findings suggest that the ICD criteria appeared to be more stringent than the DSM criteria in diagnosing GD (26, 27). For instance, Ko et al. analyzed the diagnostic validity and utility of IGD (DSM-5) and GD (ICD-11) through the empirical data, psychiatrists conducted diagnostic interviews with 69 subjects with IGD based on the DSM-5 IGD criteria, and only 44 participants with IGD (63.8%) fulfilled the criteria for GD (26).

Up until now, only a few studies have been involved in the development of standard questionnaires for the assessment of GD based on ICD-11 criteria (28, 29). Therefore, the current study aimed to develop a screening self-assessment instrument named the Gaming Disorder Symptom Questionnaire (GDSQ) to assess GD among adolescents based on ICD-11 criteria. First, we developed the GDSQ with the GD symptoms listed in ICD-11, and we borrowed the setting style of response options from the classic screening tool Alcohol Use Disorders Identification Test (30) to promote the understandability and usability of GDSQ. Then, we validated GDSQ with a large sample of Chinese adolescents aged 12–18 years. Given that all items were based on ICD-11 criteria and developed by psychiatrists and clinical psychologists with expertise in behavioral addiction, we hypothesized that the GDSQ would be a valid and reliable screening tool to assess GD.

## Methods

### Study Population and Procedure

A stratified cluster random sampling method was used to select three urban areas in Xinjiang Uygur Autonomous Region, China. These cities included Urumqi, Kashi, and Bole. In each area, two junior middle school and two senior middle school and, in each school, four classes were selected from each grade (grade 1–3 of junior middle school and grades 1–3 of senior middle school). The classes were randomly selected in each school. The students involved in the survey were aged 12–18 years old. The survey was carried out in the classrooms of the recruited classes. After the explanation of the purpose and requirements of the study, the researchers emphasized the voluntariness of the survey and then delivered the informed consent form to the parents of each adolescent. Electronic informed consent was obtained from each student after parental informed consent had been obtained. After that, the questionnaires were distributed to the students who participated in the survey.

The data in the current survey are only a part of a big set of studies that contained multiple questionnaires that need about 30 min to complete. During the filling process, the researchers answered promptly subjects' questions about the survey. The period of the data collection spanned from October 2020 to November 2021. Next, two psychiatrists assessed whether the subjects were in a high-risk GD group based on factors such as impaired control over gaming behavior, the priority of gaming over other interests and daily activities, and the continuation or escalation of gaming activities despite negative consequences.

### Ethics

Informed consent was obtained from all target participants and their parents or legal guardians. The ethical approval for this study was obtained from the Ethics Committee of the Second Xiangya Hospital of Center South University (ID: 2019-S454).

### Measures

#### Sociodemographics and Gameplay Habits

Socio-demographics information included age, gender, race, and family structure (being an only child or having siblings). Additional questions about gaming habits included starting age for gameplay, devices used to play games, preferred games, hours of game-related per week (online games, stand-alone games, and game video watching), and the amount of money spent on games per month over the past 12 months.

#### Video Gaming Dependency Scale

In the current study, the VGDS was used to assist in the development and psychometric validation process of the GDSQ as a concurrent measure of GD. IGD was assessed using the Chinese version of the VGDS (see Supplementary Table 1), which is abbreviated as CSAS in the German version “Computerspielabhängigkeitsskala” and was adapted by Rehbein et al. (31) from a previous instrument (KFN-CSAS-II). This instrument is the 18–descriptive item scale, with every two items representing one of the nine DSM-5 IGD criteria. Each item was rated on a four-point Likert scale (1 = strongly disagree, 2 = somewhat disagree, 3 = somewhat agree, and 4 = strongly agree) to evaluate the symptom severity of the subject's gaming behavior within the last 12 months. According to the DSM-5 recommendations for IGD, a criterion was endorsed if at least one of the two items was answered with “strongly agree”. The subjects who endorsed five or more of the nine symptom criteria were considered for IGD. The VGDS was validated in Chinese adolescents and young adults (32). In this study, the VGDS presented an excellent internal consistency of Cronbach's α with 0.968.

#### Gaming Disorder Symptom Questionnaire

The GDSQ was developed based on the diagnostic guidelines of ICD-11 GD. Initially, we had 24 items (see Table 1) after consulting with an expert panel to ensure content validity. The panel meeting was composed of psychiatrists and clinical psychologists with expertise in behavioral addiction. Every eight of the 24 items embodied one of the three symptoms of GD (i.e., impaired control, increasing priority to gaming, and continuing playing games despite the negative consequences). The subjects were asked to respond about the frequency of the event or situation described in the items within the last 12 months on a five-point Likert scale (0 = never, 1 = less than monthly, 2 = monthly, 3 = weekly, and 4 = almost daily). For example, “Once I start playing the game, it is hard to stop” was one item in the dimension of impaired control. The Chinese version of GDSQ is shown in Supplementary Table 2.

**Table 1:** 24 items parameters of the GDSQ, endorsement of single items (*N* = 7, 790).^a^

**ICD-11 criteria**	**Item number in pilot version**	**Item number in final version**	**Item content**	**% Endorsing each rating**				
				**0 (%)**	**1 (%)**	**2 (%)**	**3 (%)**	**4 (%)**
Impaired control	1	1	I will turn on games uncontrollably sometimes.	3,013 (38.7)	1,516 (19.4)	899 (11.5)	2,047 (26.3)	315 (4.0)
	2	2	When I see or call to mind something about game, I can't help playing the game for a while.	3,786 (48.6)	1,558 (20.0)	833 (10.7)	1,327 (17.0)	286 (3.7)
	3	3	When the devices for playing games is in my sight, I would want to turn it on to play.	4,018 (51.6)	1,448 (18.6)	759 (9.7)	1,219 (15.6)	346 (4.4)
	4	4	Once I start playing the game, it is hard to stop.	4,425 (56.8)	1,210 (15.5)	761 (9.8)	1,086 (13.9)	308 (4.0)
	5		Even at inappropriate occasions or times, I still turn on the game and play for a while.	5,749 (73.8)	909 (11.7)	421 (5.4)	579 (7.4)	132 (1.7)
	6		I experience some discomfort (e.g., decreased vision, dizziness, muscle stiffness, or wrist pain) after high-intensity game playing.	5,192 (66.6)	1,283 (15.8)	590 (7.6)	622 (8.0)	148 (1.9)
	7	5	I intend to play games less recently, but actually there is no reduction in playing time.	4,944 (63.5)	1,183 (15.2)	630 (8.1)	859 (11.0)	174 (2.2)
	8	6	I play longer or more often than the upper limits I set for my game playing.	4,987 (64.0)	1,142 (14.6)	660 (8.5)	817 (10.5)	184 (2.4)
Increasing priority	9		If my time is scheduled by myself, I play games first and put other things on the back burner.	4,774 (61.3)	1,253 (16.1)	673 (8.6)	855 (11.0)	235 (3.0)
	10	7	To play games as soon as possible, I am perfunctory in the everyday things that I have to do.	4,930 (63.3)	1,248 (16.0)	649 (8.3)	748 (9.6)	215 (2.8)
	11	8	I miss regular meals or sleep time because of playing games.	5,483 (60.4)	1,006 (13.0)	499 (6.4)	638 (8.2)	164 (2.1)
	12	9	When I play games, I pay no attention to my personal hygiene.	6,161 (79.1)	694 (8.9)	346 (4.4)	450 (5.8)	139 (1.8)
	13	10	If others' demands occupy my game time, I feel upset.	5,014 (64.4)	1,229 (15.8)	626 (8.0)	679 (8.7)	242 (3.1)
	14	11	Although some activities provoke my interest, I refuse to participate in them because they will delay playing games.	6,060 (77.8)	759 (9.7)	372 (4.8)	462 (5.9)	137 (1.8)
	15	12	I feel that the things people are excited to talk about are not as interesting as games.	6,035 (77.5)	783 (10.0)	357 (4.6)	467 (6.0)	148 (1.9)
	16	13	To play games, I cancel or postpone the leisure activities in my plan.	5,974 (76.7)	876 (11.3)	371 (4.8)	434 (5.6)	135 (1.7)
Continued use despite the occurrence of negative consequences	17a	14a	Because of playing games, I don't have enough time and energy to get the right things done.	4,877 (62.6)	1,604 (20.6)	506 (6.5)	650 (8.3)	153 (2.0)
				No	Yes			
	17b	14b	(if 14a score ≥ 1)^b^ After the aforementioned situation, I continue to play games whenever there is a chance.	6,878 (88.3)	912 (11.7)			
				0 (%)	1 (%)	2 (%)	3 (%)	4 (%)
	18a	15a	Playing games interferes with my work or learning tasks that I should complete.	5,226 (67.1)	1,376 (17.7)	469 (6.0)	579 (7.4)	140 (1.8)
				No	Yes			
	18b	15b	(if 15a score ≥ 1)^b^ After the aforementioned situation, I continue to play games whenever there is a chance.	6,941 (89.1)	849 (10.9)			
				0 (%)	1 (%)	2 (%)	3 (%)	4 (%)
	19a	16a	Because of playing games, my performance in homework, academics, or work does not match my ability.	5,369 (68.9)	1,211 (15.5)	531 (6.8)	511 (6.6)	168 (2.2)
				No	Yes			
	19b	16b	(if 16a score ≥ 1)^b^ After the aforementioned situation, I continue to play games whenever there is a chance.	6,946 (89.2)	844 (10.8)			
				0 (%)	1 (%)	2 (%)	3 (%)	4 (%)
	20a	17a	Because I play games, my family members or friends express their disappointment with me, argues with me, or become distant from me.	5,899 (75.7)	948 (12.2)	346 (4.4)	450 (5.8)	147 (1.9)
				No	Yes			
	20b	17b	(if 17a score ≥ 1)^b^ After the aforementioned situation, I continue to play games whenever there is a chance.	7,035 (90.3)	755 (9.7)			
				0 (%)	1 (%)	2 (%)	3 (%)	4 (%)
	21a	18a	I have common topics only with people who play games. I don't know what to say to someone who does not play games.	6,666 (85.6)	457 (5.9)	206 (2.6)	330 (4.2)	131 (1.7)
				No	Yes			
	21b	18b	(if 18a score ≥ 1)^b^ After the aforementioned situation, I continue to play games whenever there is a chance.	7,252 (93.1)	538 (6.9)			
				0 (%)	1 (%)	2 (%)	3 (%)	4 (%)
	22a	19a	I am worried about my future because I play games too much.	5,937 (76.2)	810 (10.4)	375 (4.8)	447 (5.7)	221 (2.8)
				No	Yes			
	22b	19b	(if 19a score ≥ 1)^b^ After the aforementioned situation, I continue to play games whenever there is a chance.	7,094 (91.1)	696 (8.9)			
				0 (%)	1 (%)	2 (%)	3 (%)	4 (%)
	23a	20a	I have negative feelings after playing games (e.g., guilt or regret).	5,673 (72.8)	1,009 (13.0)	433 (5.6)	479 (6.1)	196 (2.5)
				No	Yes			
	23b	20b	(if 20a score ≥ 1)^b^ After the aforementioned situation, I continue to play games whenever there is a chance.	7,083 (90.9)	707 (9.1)			
				0 (%)	1 (%)	2 (%)	3 (%)	4 (%)
	24a	21a	I experience sustained negative impacts from game playing on my health (e.g., weight gain, sleeping problems, neck and shoulder damage).	6,149 (78.9)	812 (10.4)	296 (3.8)	373 (4.8)	160 (2.1)
				No	Yes			
	24b	21b	(if 21a score ≥ 1)^b^ After the aforementioned situation, I continue to play games whenever there is a chance.	7,133 (91.6)	657 (8.4)			

*GDSQ, Gaming Disorder Symptom Questionnaire; ICD-11, 11th revision of the International Classification of Diseases*.

*Five-point Likert scale: “0 = never, ” “1 = less than monthly, ” “2 = monthly, ” “3 = weekly, ” “4 = almost daily”*.

a *Instructions: These question will ask you about your actual gaming-related activity during the past year (i.e., last 12 months)*.

b *Instructions: The number in parentheses shows the item number in the final version*.

It should be noted that each of the items reflecting “continuing playing games despite the negative consequences” had two-round responses. The first question was asked about the frequency of game playing. If the subjects respond with “0” (i.e., never), then there is no need to enter the second question. However, if the response is “1” (i.e., less than monthly) or more frequent, then the subjects need to respond to the second question about whether they will continue playing games or not. The options for the second question are dichotomous (0 = no, 1 = yes). The score of the item was calculated by multiplying the point of the first question with the point of the second question. For example, the first question of one item for the dimension of “continued use despite the occurrence of negative consequences” is “Because of playing games, I don't have enough time and energy to get the right things done”, and the second question is “After the aforementioned situation, I continue to play games whenever there is a chance”.

### Statistical Analysis

#### Missing Data

Among all 7,901 participants, 7,790 (98.60%) adolescents answered all items of the questionnaires and included in this study. A total of 111 (1.40%) adolescents were excluded from the analysis due to missing information in GDSQ.

#### Statistical Procedures

As the sample size for this study was sufficiently large, SPSS 25.0 was used to divide the sample into two separate data files. The first data file contained 3,871 samples and was used for exploratory factor analysis (EFA). The second data file contained 3,919 samples and was used for the confirmatory factor analysis (CFA). The full sample was used for descriptive statistical analysis and reliability estimation. Retest sample: 554 students from the first sample were retested 8 weeks apart. It was analyzed using MPLUS 8.3 for the CFA and SPSS 25.0 for Windows for the remaining analysis. A significance level of 0.05 was adopted for all statistical tests.

The chi-square (χ^2^) values were applied to detect the differences between the model's implied variance–covariance matrix and the observed variance–covariance matrix. The comparative fit index (CFI) was used to compare the hypothesized model with the null hypothesis (33–35). The CFI is also one of the most robust indicators (36). The Tucker–Lewis Index (TLI) is a relative goodness of fit indices. In addition to evaluating the model from the perspective of model fitting, the fitting degree of the model can also be evaluated from the size of the residual error, and then, the fitting situation of the model can be evaluated. The standardized root mean square residual (SRMR) is one of the indicators for the direct evaluation of residual error. The model fit was also assessed by the root mean square error of approximation (RMSEA). McDonald and Ho recommended that the model with a RMSEA < 0.08 as an acceptable one and < 0.05 as a good lone. Model goodness of fit was assumed according to the following criteria: RMSEA < 0.05 (35), SRMR < 0.08, TLI > 0.95, and CFI > 0.95 (36).

The GDSQ's ability to distinguish between non-disordered and disordered gamers was evaluated using a receiver characteristic curve (ROC) analysis. To achieve this goal, the GDSQ scores were compared against the standard according to ICD-11 related VGDS items. In this study, we also applied to defined 95% confidence intervals (CI). The Youden Index was calculated by sensitivity and specificity. The diagnostic efficacy of GDSQ was measured by the area under the ROC curve (AUC).

## Results

### Sample's Characteristics

The demographic characteristics are shown in Table 2. The mean age of first gameplay was 9.48 years (SD: 2.68). With regard to the gaming platforms preference, the majority of the participants (*n* = 3,803, 48.8%) reported playing on smartphones. The most genre played were multiplayer online battle arena (MOBA) games (*n* = 2,266, 29.1%), followed by first-person shooters (FPS) and counter-strike: global offensive (CS: GO) (*n* = 1,231, 15.8%). The average amount of money spent on gaming was 44.92 CNY (Chinese Yuan) per month (SD: 386.51). In addition, a large amount of gamers was reported spending more than 16 h per week on playing online games (*n* = 441, 5.6%), stand-alone games (*n* = 211, 2.7%), and game video watching (*n* = 224, 2.8%), respectively.

**Table 2:** Socio-demographic and gameplay characteristics of the sample (*N* = 7,790).

**Variables**		**Mean ±SD or *N* (%)**
Age, years		14.99 ± 1.65
Gender	Male	3,742 (48)
	Female	4,048 (52)
Middle school stage	Junior middle school	3,467 (44.5)
	Senior middle school	4,323 (55.5)
Ethnicity	Han nationality	5,943 (76.6)
	Other ethnic minority	1,847 (23.4)
Family structure	Being an only child	3,815 (49.0)
	Having siblings	3,975 (51.0)
Weekly gameplay	0 h	2,505 (32.2)
	<2 h	2,601 (33.4)
	Between 2 and 4 h	1,102 (14.1)
	Between 4 and 8 h	770 (9.9)
	Between 8 and 16 h	371 (4.8)
	Between 16 and 32 h	204 (2.6)
	Between 32 and 64 h	111 (1.4)
	Between 64 and 128 h	70 (0.9)
	More than 128 h	56 (0.7)
Gaming device preference	Smartphones	3,803 (48.8)
	Personal computers	445 (5.7)
	Tablets	349 (4.5)
	Portable gaming devices	21 (0.7)
Game genre preference	MOBAs	2,266 (29.1)
	FPSs, CS: GOs	1,231 (15.8)
	Other games	1,917 (24.6)

### Factor Structure

An EFA using the Principal Axis Factoring extraction method with principal oblique rotation on the 24 items of the GDSQ was performed on the whole sample (*n* = 7,790) to examine its factorial structure and construct validity. The three principal components to be extracted were determined by the convergence of the scree plot in combination with the tendency for Kaiser's criterion. The scree plot of the GDSQ-21 by 21-factor analysis (*n* = 3,871) is shown in Supplementary Figure 1.

Items with factor loadings >0.45 and/or parallel loadings <0.20 were retained. After the first rotation, three items [“Even at inappropriate occasions or times, I still turn on the game and play for a while, ” “I experience some discomfort (e.g., decreased vision, dizziness, muscle stiffness, or wrist pain) after high-intensity game playing, ” and “If my time is scheduled by myself, I play games first and put other things on the back burner”] were removed due to these three items appeared to be a cross-load problem. The appropriateness for conducting the EFA was confirmed by the Kaiser–Meyer–Olkin criterion value (KMO = 0.970) used for the suitability of the date and the Bartlett's test result of sphericity (χ^2^ = 80,321.74, *p* < 0.001). As shown in Table 3, component loadings for each item ranged between 0.689 (item 18) and 0.846 (item 11). The three factors that were extracted after six iterations explained 76.04% of the total variance. Moreover, the 21 items of the GDSQ were retained in the model for subsequent analyses (i.e., CFA) to ensure optimal construct validity.

**Table 3:** Summary of the results from the EFA on the GDSQ-21 items obtained from the sample (*n* = 3,871).

**Item in GDSQ-21**	**Factor loadings^a^,^b^,^c^**			**Communalities**		
	**Factor 1**	**Factor 2**	**Factor 3**	**Extraction**	**Corrected item-total correlation**	**Cronbach's α if item deleted**
1	**0.826**	0.149	0.175	0.735	0.677	0.964
2	**0.838**	0.256	0.218	0.817	0.768	0.962
3	**0.818**	0.297	0.211	0.801	0.775	0.962
4	**0.725**	0.378	0.260	0.737	0.795	0.962
5	**0.665**	0.448	0.288	0.725	0.813	0.961
6	**0.621**	0.500	0.284	0.716	0.815	0.961
7	0.547	**0.590**	0.322	0.751	0.847	0.961
8	0.422	**0.675**	0.324	0.739	0.823	0.961
9	0.297	**0.766**	0.332	0.786	0.806	0.961
10	0.455	**0.656**	0.276	0.713	0.806	0.961
11	0.284	**0.809**	0.332	0.846	0.824	0.961
12	0.293	**0.784**	0.351	0.823	0.824	0.961
13	0.278	**0.798**	0.355	0.841	0.826	0.961
14	0.262	0.360	**0.728**	0.728	0.770	0.962
15	0.238	0.324	**0.780**	0.770	0.764	0.962
16	0.232	0.327	**0.801**	0.802	0.774	0.962
17	0.188	0.312	**0.766**	0.719	0.720	0.963
18	0.138	0.347	**0.747**	0.689	0.697	0.963
19	0.214	0.159	**0.821**	0.746	0.677	0.963
20	0.221	0.152	**0.809**	0.727	0.671	0.963
21	0.203	0.236	**0.812**	0.757	0.710	0.963

*Greatest loadings on each factor are bolded; GDSQ-21, Gaming Disorder Symptom Questionnaire-21; EFA, Exploratory Factor Analysis*.

*GDSQ-21 factor 1 = impaired control, GDSQ-21 factor 2 = increasing priority, and GDSQ-21 factor 3 = continued use despite the occurrence of negative consequences*.

a *Percentage of variance explained by three factors = 76.04%*.

b *After six iterations, it was possible to extract three factors from the EFA*.

c *Cronbach's = 0.964*.

**Figure 1:**
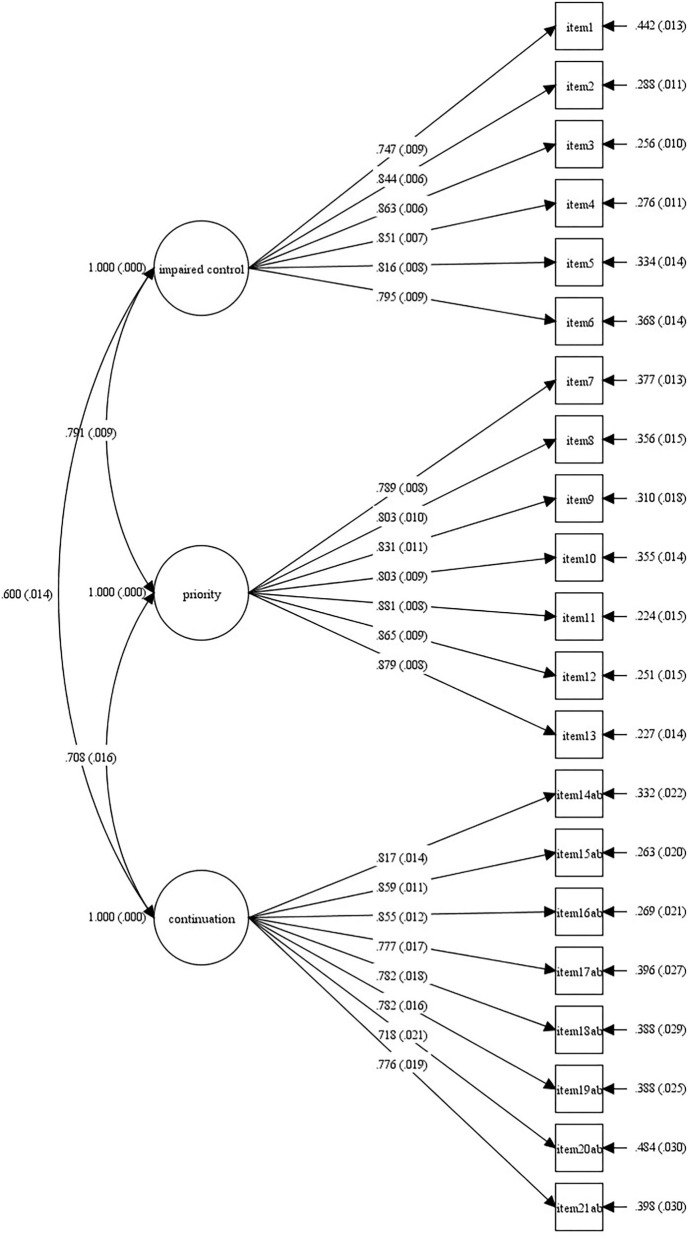
Graphical summary of CFA results obtained from the 21 items of the GDSQ-21 (*N* = 3,919). GDSQ-21, Gaming Disorder Symptom Questionnaire-21; CFA, confirmatory factor analysis.

The goodness of fit of the unidimensional model of GDSQ in EFA was assessed using the conventional fit indices. The results showed an overall good fit to the data. The χ^2^ was 1,219.11 (*p* < 0.001) with CFI of 0.958, TLI of 0.951, RMSEA of 0.038, and SRMR of 0.039, indicating an acceptable fit. As shown in Figure 1, the CFA results showed statistically significant factors (*p* < 0.05) for the 21 items of the GDSQ (GDSQ-21).

### Criterion-Related Validity and Reliability

The results of the eight multiplications are summed. By summing up the responses to the 21 items, the total score was calculated to obtain a possible maximum score of 84. To further assess the validity and reliability of the GDSQ-21, the total scores obtained by participants on the VGDS and GDSQ-21 were associated (*r* = 0.781, *p* < 0.001). The GDSQ-21 was associated with self-reported weekly gaming time (online, stand-alone, and game video watching) (*r* = 0.619, 0.514, and 0.504, respectively, *p*s < 0.001). The GDSQ-21 was also associated with the monthly amount of money spent on games (Spearman's ρ = 0.338, *p* < 0.001). As shown in Table 4, the results obtained suggest that the GDSQ-21 is strongly positively associated with VGDS, and moderately correlated with the self-reported weekly gaming time and the monthly amount of money spent. The Spearman–Brown split-half reliability of GDSQ-21 was 0.98, and the test–retest reliability was 0.71. Cronbach's α of the GDSQ-21 was 0.964 in this analysis, which showed the scale's good internal consistency. The Cronbach's α for the three factors were 0.929, 0.950, and 0.948, respectively.

**Table 4:** Correlations between GDSQ-21 and other related measures.

**Construct**	***r*** **/ρ**	***p***
VGDS sum score	0.781	<0.001
Weekly online gaming time (h)	0.619	<0.001
Weekly stand-alone gaming time (h)	0.514	<0.001
Weekly game video watching time (h)	0.504	<0.001
Money spent on gaming/month (CNY)	0.338	<0.001

*GDSQ-21, Gaming Disorder Symptom Questionnaire-21; r, Pearson's correlation coefficient; ρ, Spearman's correlation; VGDS, Video Gaming Dependency Scale; CNY, Chinese Yuan*.

### Cutoff Points of the GDSQ-21 for GD

**Figure 2:**
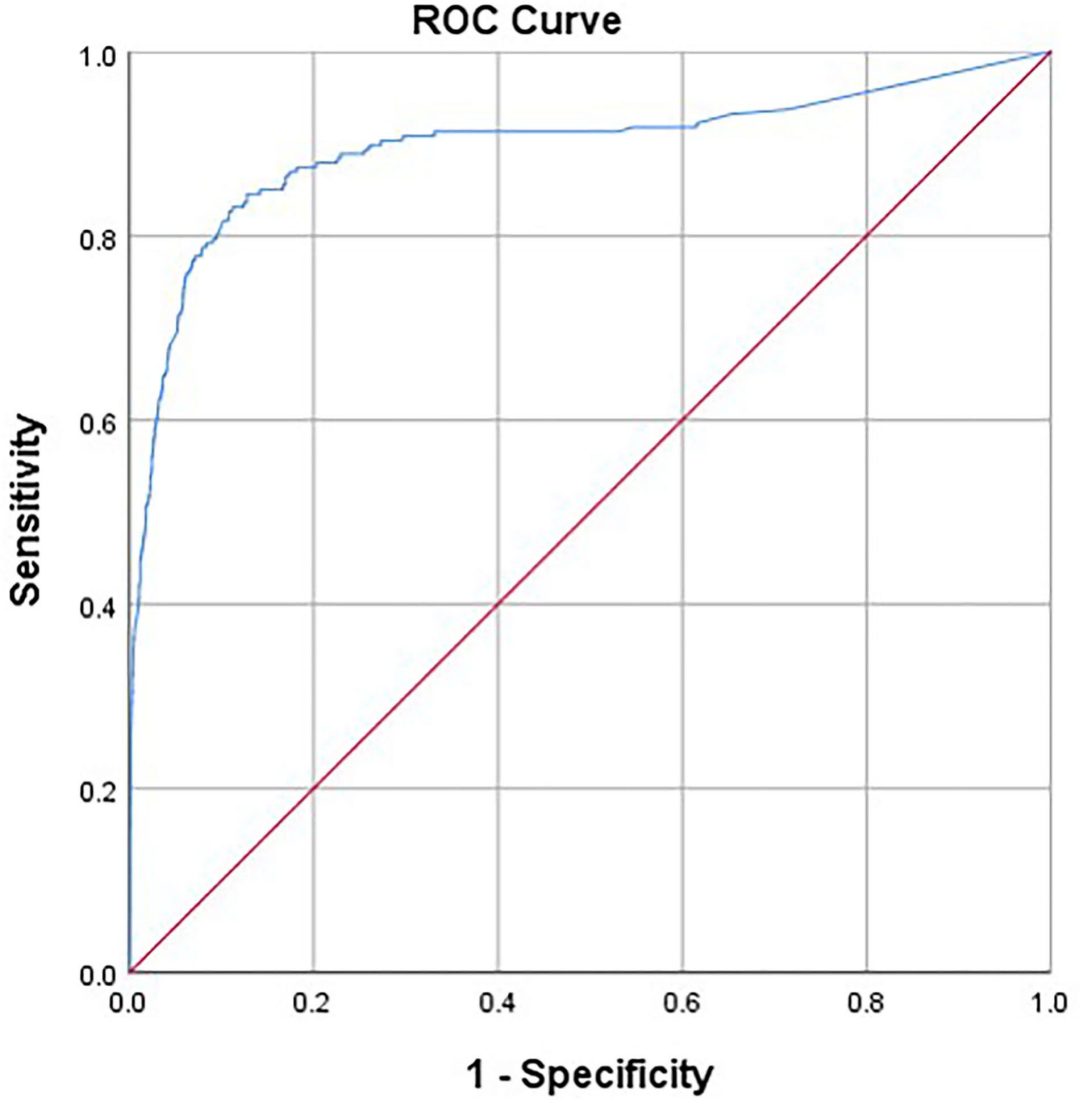
Area under the ROC curve of the GDSQ-21 for diagnosis (AUC, 0.896). ROC, receiver operating characteristic; AUC, area under the ROC curve.

As shown in Figure 2, by the Youden Index, the optimal cutoff for the overall score was 61.5 with the curve (AUC) of 89.6% (95% CI = 86.6–92.7), sensitivity of 83.1%, and specificity of 88.8%. Factor 1 had a cutoff value of 13.5 with the curve (AUC) of 86.6% (95% CI = 83.5–90.0), specificity of 78.3%, and sensitivity of 86.7%. Factor 2 had a cutoff value of 10.5 with the curve (AUC) of 89.2% (95% CI = 86.3–92.1), specificity of 78.7%, and sensitivity of 89.3%. Factor 3 had a cutoff value of 3.5 with the curve (AUC) of 86.0% (95% CI = 82.6–89.4), specificity of 77.3%, and sensitivity of 89.4%. Finally, the cutoff ≥ 14 is applied for factor 1, ≥11 for factor 2, ≥4 for factor 3, and ≥62 for the whole scale. Participants who met each dimension and total score were classified with GD. The prevalence of GD was estimated at 2.27% in the period of the past 12 months. As shown in Table 5, there was a significant difference between GD and No-GD adolescents. For instance, the GD adolescents reported more weekly gaming time than the No-GD adolescents.

**Table 5:** Grouping of GD and No GD according to ROC curve cutoffs (mean ± SD).

**Variables**	**GD (*n* = 177)**	**No GD (*n* = 7,673)**	***t***	***p***
Age, years	15.65 ± 1.73	14.98 ± 1.65	5.19	<0.001
GDSQ-21 factor 1 score	21.07 ± 2.65	5.50 ± 5.94	73.966	<0.001
GDSQ-21 factor 2 score	23.82 ± 3.38	3.27 ± 5.40	78.369	<0.001
GDSQ-21 factor 3 score	24.90 ± 4.43	1.31 ± 3.89	70.158	<0.001
VGDS sum score	59.82 ± 10.32	25.18 ± 10.32	44.155	<0.001
Weekly online gaming time (h)	5.18 ± 2.01	2.38 ± 1.53	18.390	<0.001
Weekly stand-alone gaming time (h)	4.8 ± 2.23	1.84 ± 1.20	17.614	<0.001
Weekly game video watching time (h)	4.74 ± 2.35	1.75 ± 1.19	16.899	<0.001
Money spent on gaming/month (CNY)	406.14 ± 1,296.80	35.34 ± 325.81	3.356	<0.001

*GD, Gaming Disorder; No-GD, No Gaming Disorder; ROC, Receiver Operating Characteristic; GDSQ-21, Gaming Disorder Symptom Questionnaire-21; GDSQ-21 factor 1 = impaired control, GDSQ-21 factor 2 = increasing priority, and GDSQ-21 factor 3 = continued use despite the occurrence of negative consequences; VGDS, Video Gaming Dependency Scale; CNY, Chinese Yuan*.

## Discussion

To the best of our knowledge, this study is the first study to introduce a screening tool for assessing the ICD-11 diagnostic guidelines for GD in adolescents in China. GDSQ-21 was successfully validated in a sample of adolescents as an assessment tool with excellent internal consistency and criterion validity. The instrument covers the three symptoms for IGD/GD, and the concrete manifestations of these symptoms are referred to in detail (i.e., impaired control, increasing priority to gaming, and continued use despite the occurrence of negative consequences) and functional impairment. Thus, in addition to exhibiting psychometrically robust properties, it is an easy-to-use screening instrument that can be used by medical and non-medical institutions to distinguish non-disordered and disordered gamers.

In respect of the test reliability and validity, the GDSQ-21 appears to be a reliable and valid measure for assessing GD. The statistically significant positive associations were found among the GDSQ-21, weekly gaming hours (online, stand-alone, and video watching), average monthly gaming charge, and the VGDS test, providing empirical evidence for the validity of the test. Moreover, results from the EFA and CFA support the population cross validity of the GDSQ-21 as it was shown that the three-factor solution found in the EFA (i.e., sample 1) was also replicated and confirmed in the CFA (i.e., sample 2). Furthermore, the instrument was highly reliable in all samples as the Cronbach's α values were very high, suggesting that the GDSQ-21 measurement is reliable and accurate in detecting changes in GD levels.

Game duration and the game genres preference were significantly correlated with GD. GDSQ-21 sum scores were also associated with weekly playtime and average monthly spending on games. Previous studies have reported that spending a lot of time and money is a predictor of IGD (37). As seen in the game devices, more and more players are turning to smartphone games and table games. The current study found that smartphones may be more addictive than other devices for pathological gamers, and this finding is supported by Christian Montag's study (38, 39). In terms of game genres, the characteristics of different types of games attract different gamers, whereas the occurrence, symptoms, and negative consequences of GD are related to the genres of the game (6, 13). In general, complex, endless, and socially driven game types are more likely to have GD (40). It is worth noting that future studies should focus on different risky situations, as different situations require the adaptation of (early) intervention methods for optimal recovery of GD.

In the ROC analysis, the AUC was 89.6%, indicating strong discriminatory power. With adequate sensitivity and specificity, the GD was effectively distinguished from no GD. However, it is important to further examine whether the cutoff point of 62 (of a total score of 84) can distinguish disordered gamers from non-disordered among different populations, such as adults and individuals from other regions or countries.

Compared to the DSM-5 diagnostic criteria for IGD, the ICD-11 definition of GD may be more concise and research-based, highlighting the most central symptom presentation. Studies have found different rates of GD in screening between the DSM-5–based IGD criteria and the ICD-11 GD guidelines. For instance, the 12-month prevalence of IGD in Chinese adolescents was 2.9% according to DSM-5 criteria (22) and 2.27% in currently study.

Pontes and Mark (41) have also reported various inconsistencies and psychometric weaknesses in IGD instrumentation, but the GDSQ-21 instrument is considerable and has sufficiently sufficient reliability and validity. Its three factors take into account the size and intensity of the range of problematic game performance, and their items are not independent but interrelated. All items were good construct indicators due to all factor loadings being statistically significant and relatively high.

Although the available IGD instruments are still applicable measures based on the DSM-5 framework, the GDSQ-21 is a psychometric instrument developed under the new ICD-11 framework that will yield fundamental clinical and diagnostic differences between GD-based psychometric assessment instruments. The GDSQ-21 scale has excellent reliability and validity, and the theoretical concepts and connotations based on it are consistent with international standards.

Although the present study provides unique information about the ICD-11 criteria for GD, the limitations should be considered. First, given that adolescents are the most vulnerable group of GD, all participants in this study were adolescent students (aged 12–18 years). Thus, the current samples was not fully representative, and findings from current study should be cautiously interpreted in terms of its generalizability. Future studies should include adults and children to further confirm the robustness of the GDSQ-21. Second, the GD was assessed by the self-report questionnaires but not by professional clinical interviews and diagnoses. Future research in the field should compare clinically diagnosed sample with actual GDSQ-21 test scores.

## Conclusions

In conclusion, the value of the GDSQ-21 as a GD screener for adolescents is evidenced by the current findings. The GDSQ-21 has excellent internal consistency reliability and criterion validity in a representative sample of adolescent game players. Furthermore, its three-factor structure supports the ICD-11 new diagnostic concept of GD, regarding persistent gaming behavior, impaired control over gaming, and functional impairment due to gaming for at least 12 months in most instances. Findings from this study recommend the use of the GDSQ-21 as a screening tool to assess GD, which can assist non-medical providers to screen people with GD.

## Data Availability Statement

The original contributions presented in the study are included in the article/Supplementary Material, further inquiries can be directed to the corresponding authors.

## Ethics Statement

The studies involving human participants were reviewed and approved by the Ethics Committee of the Second Xiangya Hospital of Center South University (ID: 2019-S454). Written informed consent to participate in this study was provided by the participants' legal guardian/next of kin.

## Author Contributions

WH, MY, and LZ designed the assessment tools and study protocol. LZ and YC collected the data. LZ, YL, and TL performed the literature review, statistical analysis, and wrote the first draft. YL and TL commented on the manuscript. All authors interpreted the data, critically reviewed the content, and approved the submitted version.

## Funding

This study was supported by the Non-profit Central Research Institute Fund of the Chinese Academy of Medical Sciences (No. 2019HY320001).

## Conflict of Interest

The authors declare that the research was conducted in the absence of any commercial or financial relationships that could be construed as a potential conflict of interest.

## Publisher's Note

All claims expressed in this article are solely those of the authors and do not necessarily represent those of their affiliated organizations, or those of the publisher, the editors and the reviewers. Any product that may be evaluated in this article, or claim that may be made by its manufacturer, is not guaranteed or endorsed by the publisher.
