# Supplementary material for: Gaming Disorder Symptom Questionnaire: The Development and Validation of a Screening Tool for ICD-11 Gaming Disorder in Adolescents
Source: Front Psychiatry. 2022 Mar 24;13:848157. doi: 10.3389/fpsyt.2022.848157 (PMC8987917; doi:10.3389/fpsyt.2022.848157)
Supplement: Supplementary file 1 [file Data_Sheet_1.docx]

**S TABLE 1 |** 视频游戏依赖量表（Video Game Dependency Scale）

阅读下列描述，您是否同意它们描述了最近的一年中您的实际情况。请在1～4上选择画圈“○”做出回答。

1 = 非常不同意，2 = 有点不同意，3 = 有点同意，4 = 非常同意

| 问题 | 最近一年中 | | | |
| --- | --- | --- | --- | --- |
| 1. 即使是在没有玩游戏的时候，游戏也在我脑海里 | 1 | 2 | 3 | 4 |
| 2. 即使不玩游戏的时候，我的想法一直都围绕着游戏 | 1 | 2 | 3 | 4 |
| 3. 玩不到游戏时，我会容易发脾气或觉得不开心 | 1 | 2 | 3 | 4 |
| 4. 一段时间不玩游戏，我就开始感到不安和紧张 | 1 | 2 | 3 | 4 |
| 5. 我感到游戏对我而言越来越重要 | 1 | 2 | 3 | 4 |
| 6. 我觉得需要花越来越多的时间玩游戏才能从中得到满足 | 1 | 2 | 3 | 4 |
| 7. 我感到无法控制自己花在游戏上的时间长短 | 1 | 2 | 3 | 4 |
| 8. 我尝试减少花在游戏上的时间，但失败了 | 1 | 2 | 3 | 4 |
| 9. 频繁的游戏给我的学业或工作带来麻烦 | 1 | 2 | 3 | 4 |
| 10. 因为花太多时间玩游戏，我常常跟家人吵架或争论 | 1 | 2 | 3 | 4 |
| 11. 因为玩游戏，我对以前喜欢的活动不再那么喜欢了 | 1 | 2 | 3 | 4 |
| 12. 因为觉得游戏更重要，我放弃或者减少了其他爱好活动 | 1 | 2 | 3 | 4 |
| 13. 我常常在生活中的其他事情不顺利时去玩游戏 | 1 | 2 | 3 | 4 |
| 14. 玩游戏是我忘记现实问题的最好方式 | 1 | 2 | 3 | 4 |
| 15. 我玩游戏的实际时间比我向别人承认的要更多 | 1 | 2 | 3 | 4 |
| 16. 我对别人撒谎，隐瞒我在玩游戏上花了多少时间 | 1 | 2 | 3 | 4 |
| 17. 因为玩游戏，我的一些重要的人际关系或友谊已经受到损害，或可能受到损害 | 1 | 2 | 3 | 4 |
| 18. 我曾经冒着学业失败或丢掉工作的风险玩游戏 | 1 | 2 | 3 | 4 |

**S TABLE 2 |** 游戏障碍症状问卷Chinese version of the Gaming Disorder Symptom Questionnaire

请回忆您最近一年中与玩游戏相关的实际情况，在每一项描述后面选择最能描述实际频率或时间间隔的选项，将相应分数填到空白方格里。

| 初版  编号 | 终版  编号 | 问题 | 0 | 1 | 2 | 3 | 4 | 最近  一年中 |
| --- | --- | --- | --- | --- | --- | --- | --- | --- |
| 1 | 1 | 我有时似乎会不由自主地打开游戏 | 从来  没有 | 不到  每月1次 | 每月  1次 | 每周  1次 | 几乎每天 |  |
| 2 | 2 | 看到或想到游戏相关的内容后，我就忍不住要玩一会游戏 | 从来  没有 | 不到  每月1次 | 每月  1次 | 每周  1次 | 几乎每天 |  |
| 3 | 3 | 玩游戏的设备在我眼前，我就会想打开玩游戏 | 从来  没有 | 不到  每月1次 | 每月  1次 | 每周  1次 | 几乎每天 |  |
| 4 | 4 | 我一开始玩游戏就难以停下来 | 从来  没有 | 不到  每月1次 | 每月  1次 | 每周  1次 | 几乎每天 |  |
| 5 |  | 在不适合玩游戏的场合或时机，我也会打开游戏玩一会 | 从来  没有 | 不到  每月1次 | 每月  1次 | 每周  1次 | 几乎每天 |  |
| 6 |  | 高强度的游戏之后，我会出现一些不适表现（例如视力下降、头晕、肌肉僵硬、或手腕酸痛） | 从来  没有 | 不到  每月1次 | 每月  1次 | 每周  1次 | 几乎每天 |  |
| 7 | 5 | 我打算最近少玩一点游戏，但实际上玩起来并没有减少 | 从来  没有 | 不到  每月1次 | 每月  1次 | 每周  1次 | 几乎每天 |  |
| 8 | 6 | 我给自己玩游戏的频率或时长设置了上限，但实际上依然超过上限 | 从来  没有 | 不到  每月1次 | 每月  1次 | 每周  1次 | 几乎每天 |  |
| 9 |  | 在时间由我自己安排的情况下，我就先玩游戏，把其他事往后排 | 从来  没有 | 不到  每月1次 | 每月  1次 | 每周  1次 | 几乎每天 |  |
| 10 | 7 | 我为了尽快去玩游戏而草草完成一些不得不做的日常事情 | 从来  没有 | 不到  每月1次 | 每月  1次 | 每周  1次 | 几乎每天 |  |
| 11 | 8 | 我因为玩游戏而错过正常的吃饭或睡觉时间 | 从来  没有 | 不到  每月1次 | 每月  1次 | 每周  1次 | 几乎每天 |  |
| 12 | 9 | 我玩起游戏来就不注意自己的个人卫生 | 从来  没有 | 不到  每月1次 | 每月  1次 | 每周  1次 | 几乎每天 |  |
| 13 | 10 | 别人要求我做的事情占用我玩游戏的时间，我就感到不高兴 | 从来  没有 | 不到  每月1次 | 每月  1次 | 每周  1次 | 几乎每天 |  |
| 14 | 11 | 一些活动引起了我的兴趣，但因为会耽误玩游戏，我就拒绝参加 | 从来  没有 | 不到  每月1次 | 每月  1次 | 每周  1次 | 几乎每天 |  |
| 15 | 12 | 别人兴致勃勃谈论的事，我认为不如游戏有意思 | 从来  没有 | 不到  每月1次 | 每月  1次 | 每周  1次 | 几乎每天 |  |
| 16 | 13 | 我原本打算要进行的休闲娱乐活动因为玩游戏而取消或推迟 | 从来  没有 | 不到  每月1次 | 每月  1次 | 每周  1次 | 几乎每天 |  |
| 17a | 14a | 玩游戏使得我没有足够的时间和精力去做好该做的正事 | 从来  没有 | 不到  每月1次 | 每月  1次 | 每周  1次 | 几乎每天 |  |
| 17b | 14b | （如果14a得分≥1）^a^发生这种情况后，只要有机会我又会玩游戏 | 否 | 是 |  |  |  |  |
| 18a | 15a | 我因为玩游戏而耽误了本该完成的工作或学习任务 | 从来  没有 | 不到  每月1次 | 每月  1次 | 每周  1次 | 几乎每天 |  |
| 18b | 15b | （如果15a得分≥1）^a^发生这种情况后，只要有机会我又会玩游戏 | 否 | 是 |  |  |  |  |
| 19a | 16a | 因为玩游戏，我的作业结果、学习成绩或工作绩效没达到与我本身能力相称的水平 | 从来  没有 | 不到  每月1次 | 每月  1次 | 每周  1次 | 几乎每天 |  |
| 19b | 16b | （如果16a得分≥1）^a^发生这种情况后，只要有机会我又会玩游戏 | 否 | 是 |  |  |  |  |
| 20a | 17a | 亲人或朋友因为我玩游戏而表示失望、与我争吵或变疏远 | 从来  没有 | 不到  每月1次 | 每月  1次 | 每周  1次 | 几乎每天 |  |
| 20b | 17b | （如果17a得分≥1）^a^发生这种情况后，只要有机会我又会玩游戏 | 否 | 是 |  |  |  |  |
| 21a | 18a | 我只与玩游戏的人有共同话题，如果对方不玩游戏，我不知道该聊些什么 | 从来  没有 | 不到  每月1次 | 每月  1次 | 每周  1次 | 几乎每天 |  |
| 21b | 18b | （如果18a得分≥1）^a^发生这种情况后，只要有机会我又会玩游戏 | 否 | 是 |  |  |  |  |
| 22a | 19a | 我因为玩游戏太多而感到自己前途堪忧 | 从来  没有 | 不到  每月1次 | 每月  1次 | 每周  1次 | 几乎每天 |  |
| 22b | 19b | （如果19a得分≥1）^a^发生这种情况后，只要有机会我又会玩游戏 | 否 | 是 |  |  |  |  |
| 23a | 20a | 玩游戏之后感到负面情绪，例如内疚或后悔 | 从来  没有 | 不到  每月1次 | 每月  1次 | 每周  1次 | 几乎每天 |  |
| 23b | 20b | （如果20a得分≥1）^a^发生这种情况后，只要有机会我又会玩游戏 | 否 | 是 |  |  |  |  |
| 24a | 21a | 我感到玩游戏对我的健康状况有持续的负面影响（例如体重变化、睡眠问题、颈肩受损） | 从来  没有 | 不到  每月1次 | 每月  1次 | 每周  1次 | 几乎每天 |  |
| 24b | 21b | （如果21a得分≥1）^a^发生这种情况后，只要有机会我又会玩游戏 | 否 | 是 |  |  |  |  |

注：^a^括号里面写的编号是最终版的条目编号


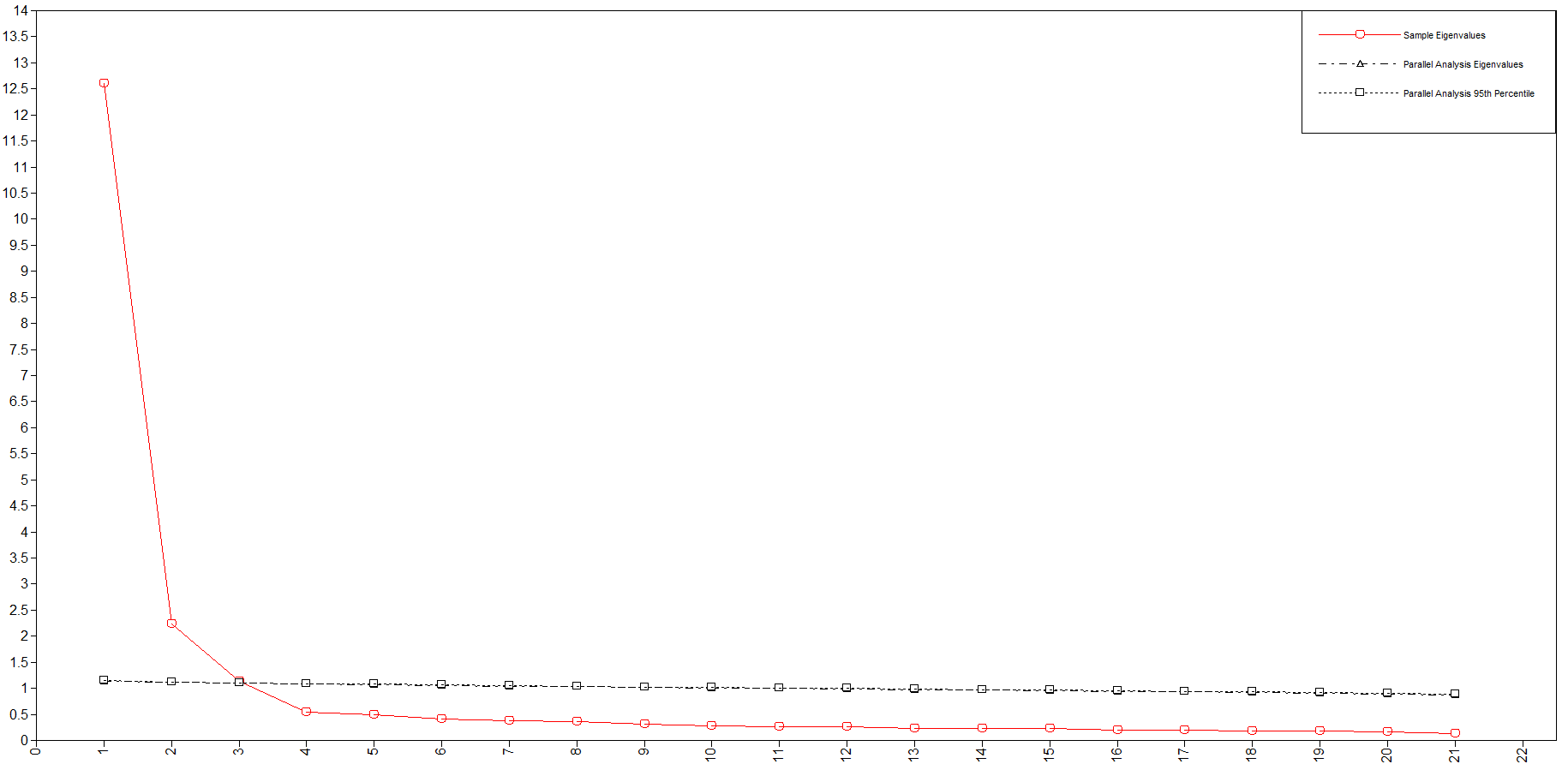


**S FIGURE 1 |** The Scree Plot of the Game Disorder Manifestation Questionnaire - 21 (GDSQ-21) by 21 factor analysis (n = 3871).
